# The effect of ruthenium oxidation on the decomposition of SiH_4_

**DOI:** 10.1039/d6cp01417h

**Published:** 2026-06-16

**Authors:** Ester Perez-Penco, Jonathon Cottom, Emilia Olsson, Roland Bliem

**Affiliations:** a Advanced Research Center for Nanolithography, Science Park 106 1098 XG Amsterdam The Netherlands r.bliem@arcnl.nl; b Institute for Theoretical Physics, Institute of Physics, University of Amsterdam, Science Park 904 1098 XH Amsterdam The Netherlands; c van der Waals–Zeeman Institute, Institute of Physics, University of Amsterdam, Science Park 904 1098 XH Amsterdam The Netherlands

## Abstract

In chemical vapor deposition, the interaction of the precursor molecule with the substrate can crucially influence the deposition process and provide pathways to tailor the growth. Here, we report mechanistic differences of Si growth from silane (SiH_4_) at pristine and oxidized surfaces of Ru(0001) in a combined *in situ* X-ray photoelectron spectroscopy (XPS) and density functional theory (DFT) study. Silane decomposition is essentially barrierless and thus very efficient on Ru(0001), where it saturates at approximately a monolayer of silicon. Similarly, silane readily decomposes on a thin intermediate phase of Ru oxide, which is structurally different from thicker RuO_2_ layers, and induces a transformation to a compound with oxidized Si and reduced Ru. In contrast, the bulk-like RuO_2_ phase is observed to be fully inert towards the decomposition of SiH_4_ at room temperature. This difference between the two oxides is suggested to originate from disorder and the availability of active sites in the thin Ru oxide layer. The surface-assisted deposition of silicon on Ru can thus be modified and inhibited using oxidation, depending on the nature of the oxide layer.

## Abstract 2

Silane (SiH_4_) decomposes efficiently on Ru(0001) and on Ru(0001) with a thin Ru oxide layer up to saturation coverages close to a single layer. The bulk-like RuO_2_ phase is fully inert towards SiH_4_ decomposition at room temperature.

## Graphical abstract

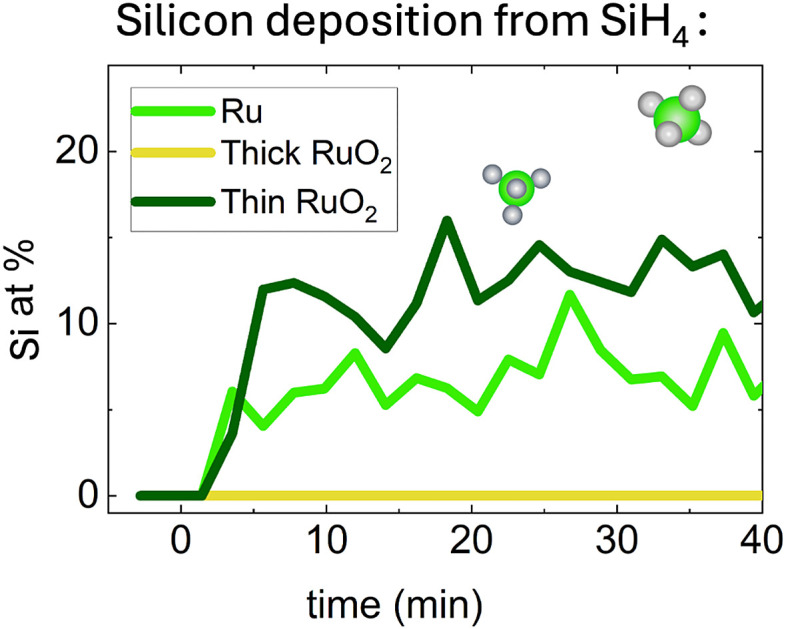

## 1 Introduction

Continued downscaling of complementary metal–oxide–semiconductor (CMOS) technology drives interconnect dimensions into the deep-nanometer regime, where line resistance and reliability of the back end of line become key performance limiters.^1,2^ In this regime, conventional Cu plus diffusion-barrier stacks suffer from strong resistivity scaling due to enhanced surface and grain-boundary scattering and the geometric penalty of thick barriers.^3^ Ruthenium has therefore emerged as a leading candidate for barrierless or linerless metallization, combining relatively low bulk resistivity with good electromigration reliability and compatibility with area-selective deposition strategies.^1–6^ Many of these process schemes inevitably expose Ru and Ru oxides to silicon-containing precursor molecules, either deliberately—in area-selective Ru growth, self-aligned barrier concepts, or silicide formation—or unintentionally, through cross-contamination in hydrogen-rich plasma environments.^7,8^ Understanding how the oxidation state of Ru controls the interaction with such precursors is therefore essential both for enabling controllable precursor-based deposition and for mitigating Si contamination in advanced interconnect modules.

The oxidation of Ru(0001) has been studied in substantial detail and provides a well-defined system for correlating structure and reactivity. The oxidation of the Ru(0001) surface is generally agreed to proceed *via* three regimes: (i) ordered chemisorbed O adlayers, (ii) thin surface-oxide structures, and (iii) bulk rutile RuO_2_ film.^9–13^ At low oxygen exposures, the surface exhibits a sequence of well-ordered chemisorbed O phases with coverages up to one monolayer, including p(2 × 2)-O, p(2 × 1)-O, (2 × 2)-3O and the dense (1 × 1)-O overlayer.^14–16^ These adlayers are now quantitatively characterized both experimentally and theoretically and constitute a robust reference for a metallic Ru(0001) surface decorated by on-surface oxygen. At intermediate oxygen coverages and higher oxygen chemical potentials, the picture is less clear: oxygen incorporation below the top Ru layer leads to subsurface O islands and the formation of oxide-like surface structures, but the detailed atomistic structure of this intermediate oxide remains an open question. Density-functional theory (DFT) predicts that an O–Ru–O trilayer is a low-energy metastable precursor and that stacking of such units provides a pathway towards a rutile-like RuO_2_ film once a critical thickness is exceeded.^11^ Subsequent real-space microscopy and diffraction have shown that oxygen-rich Ru(0001) is better described in terms of RuO_2_-derived domains, in particular RuO_2_(100)-(1 × 1) and RuO_2_(110) facets, rather than a single trilayer structure.^10,12,13,16,17^ Within this intermediate regime, disorder appears in two ways: as explicitly disordered or defect-rich oxide regions, and as structural disorder within otherwise RuO_2_-like domains, both of which have been implicated as catalytically active phases.^17,18^ At sufficiently high temperatures and oxygen pressures, the oxidation terminates in continuous RuO_2_(110) films with bulk-like rutile structure of well-defined stoichiometry.^10,12,13^

These distinct oxidation regimes of Ru(0001) are known to exhibit markedly different chemical reactivity. Chemisorbed O adlayers modulate the adsorption energies and activation barriers for small molecules, but retain many features of a metallic surface.^11,14,15^ In contrast, the formation of RuO_2_-like surface oxides introduces a transition to oxide-like electronic structure and coordinatively unsaturated cation sites, which profoundly change the kinetics and selectivity of oxidation reactions such as CO oxidation and NO_2_ activation.^10,12,13,18^ Subtle changes in oxide thickness, orientation and defect density can thus lead to large changes in reaction rates and preferred pathways on Ru-based catalysts.^10,12,13^ From the perspective of interconnect processing, this implies that small variations in surface preparation—ranging from a clean metal surface through thin defective oxides to thick RuO_2_—may qualitatively alter the interaction with reactive precursor molecules, even if the nominal substrate is “Ru(0001)” in all cases.

Silane (SiH_4_) and related SiH_*x*_ species are ubiquitous precursors for silicon-containing films in chemical vapor deposition (CVD), plasma-enhanced CVD, and plasma-assisted processes for amorphous and microcrystalline Si, dielectrics, and barrier layers.^19–24^ In the gas phase, silane decomposes only at comparatively high temperatures, with significant pyrolysis typically occurring above ∼420 °C.^25,26^ On high-surface-energy metal substrates, silane and its fragments can decompose at far lower temperatures than in the gas phase, frequently at or below room temperature, yielding Si adlayers or surface silicides along with chemisorbed hydrogen.^27–31^ For example, on Cu(111) silane adsorption at cryogenic temperatures leads to SiH_3_ fragments that decompose upon modest heating to give elemental Si and bridging H,^27,28^ while on Ni(111) and Pd(100) silane CVD produces surface alloys and silicide-like phases.^29,32^ These studies establish that reactive late transition-metal surfaces can strongly catalyze Si–H bond activation, enabling Si deposition at temperatures relevant for interconnect processing.

In contrast, the microscopic interaction of silane with Ru(0001) and its oxides is still only partially understood, despite its relevance to both contamination control and purposeful Si incorporation. An early UHV study of disilane (Si_2_H_6_) adsorption on Ru(0001) using LEED, AES and RAIRS showed that Si_2_H_6_ dissociates *via* SiH fragments to elemental Si and, upon annealing, to an ordered Ru silicide overlayer,^33^ demonstrating efficient Si–H bond activation on the clean Ru(0001) surface. Plasma-based experiments have shown that volatile Si hydrides generated by hydrogen radicals can undergo self-limiting chemisorption on Ru thin films, yielding approximately one monolayer of chemisorbed Si under near-equilibrium conditions.^7^ Together these studies suggest a strong tendency of Ru surfaces to activate Si–H bonds and accommodate Si at the surface. However, they do not resolve how the detailed oxidation state and structure of the Ru substrate—clean metal, thin defective oxide, or thick RuO_2_—control the elementary adsorption and decomposition steps of silane itself. Moreover, apart from the disilane study on Ru(0001),^33^ most prior work either relies on polycrystalline Ru, complex multi-component plasma environments,^7^ or focuses on the growth of fully oxidized SiO_2_-like films on Ru(0001),^34^ leaving a gap in mechanistic understanding of the earliest stages of SiH_*x*_ adsorption and decomposition on structurally well-defined Ru(0001) and RuO_*x*_ surfaces.

Here, we follow the deposition of Si from silane *in situ*, comparing the adsorption mechanisms and kinetics at the pristine Ru(0001) surface and oxidized Ru(0001) with different oxide thicknesses. We observe a stark contrast between the highly reactive surfaces at low oxide thicknesses and a fully inert layer of the bulk-like phase of rutile RuO_2_. While increasing oxygen coverages on Ru(0001) are predicted to block silane adsorption, high sticking and changes in the deposited Si species indicate that different adsorption mechanisms prevail in the presence of oxygen. The complex role of oxygen in the site selection and Si chemistry illustrates the profound implications of seemingly subtle surface modifications on reactive deposition and contamination control.

## 2 Methods

The study was performed on a Ru(0001) single crystal in an ultra-high vacuum (UHV) setup with a base pressure below 1.0 × 10^−9^ mbar for preparation and analysis. The sample was cleaned by Ar-ion sputtering for 35 minutes (p(Ar) = 5 × 10^−7^mbar, 1.5 kV), followed by annealing for 1 h at 700 °C in UHV using radiative heating of the back of a metal sample plate. Next, the Ru(0001) was exposed to p(O_2_) = 5 × 10^−7^ mbar for 30 minutes at 700 °C in order to remove carbon contamination. The absence of contaminants and residual oxygen was confirmed using XPS. Oxidation of the Ru(0001) was performed at 380 °C at an oxygen pressure of 1.2 × 10^−4^ mbar (Messer CAN-gas O_2_ 5.0 purity) to obtain ruthenium oxide layers of different thickness, and hence different structure: 0.4 nm of RuO_2_ (thin oxide) and 1.2 nm of RuO_2_ (mixed oxide, contains patches of thin oxide structure and rutile RuO_2_). Oxidation at the same pressure and 430 °C resulted in a 4.2 nm thick layer of rutile RuO_2_ (thick oxide/r-RuO_2_). The thicknesses were approximated from XPS peak intensities of metallic and oxidized components (Ru^4+^) of the Ru 3d level assuming a homogeneous flat layer.^35^ The same approximation is also used in the calculation of the monolayer coverage of Si on Ru. The different attenuation coefficients are corrected for with attenuation factors making use of the TPP-2M relation.^36^ The sample temperatures were measured using an N-type thermocouple and a 2-color SensorTherm M322 pyrometer (above 300 °C).

The as-prepared Ru surfaces were exposed to a mixed gas containing 1 vol% SiH_4_ (≈0.92 mol%) in Ar gas at a starting total pressure of 4 × 10^−6^ mbar and increased until a maximum total pressure of 4.0 × 10^−4^ mbar. The pressure increases are indicated by gray dashed lines in all datasets except for the thick oxide (r-RuO_2_), for which the last increase occurred at a later point in time and would have extended the *x*-axis excessively. This step is not represented within the plotted range, as it caused no observable effect on the surface. In the experiment on the effect of X-rays on the activation of silane, the gas supply was closed and allowed to reach UHV again before starting the X-ray source for each data point and re-opened only after switching off the X-ray source. The surface composition during SiH_4_ exposure was investigated using near-ambient pressure XPS with a Scienta Omicron HiPP-3 spectrometer and a monochromatic Al Kα X-ray source (1486.6 eV). The HiPP-3 analyzer was used with a 0.8 mm cone and a slit setting of 1.0 mm. Survey spectra were acquired at a pass energy of 300 eV, Ru 3d, Si 2p and O 1s spectra at 300 eV, and high-resolution spectra at 100 eV. For each pass energy, the measured XPS binding energies were shifted using the Ru metal peak as a reference. XPS peak fitting was performed using KolXPD applying Shirley backgrounds for all peaks. To ensure reproducible and comparable fitting results, constraints based on fundamental physical principles, literature values, and reference samples were systematically applied. Specifically, Ru reference spectra for the main and satellite peaks were obtained from oxygen-free and fully oxidized Ru reference samples to determine the Lorentzian and Gaussian components and, where applicable, the asymmetry parameters of the peak shapes (Voigt, Doniach-Sunjic convoluted with Gaussian). The peak shapes of all regions as well as the energy difference and area ratio (Ru 3d_5/2_ : Ru 3d_3/2_ = 1.5) of the spin–orbit-split components of Ru 3d were kept constant for all fits. The binding energies were fixed with respect to the Ru 3d metal peak, allowing for no relative variation of the individual species but small (<100 meV) changes of the reference energy between different sets of measurements. For both O 1s and Si 2p spectra, the focus was the determination of the total area of peaks emerging at *a priori* unknown binding energies and peak shapes. No constraints were applied to those peaks.

All DFT calculations were performed spin-polarized at the Γ-point using CP2K (v2023.1)^37^ with DZVP-SR-MOLOPT basis sets^38^ and GTH pseudopotentials.^39,40^ Plane-wave cutoffs of 850 Ry (CUTOFF) and 60 Ry (REL_CUTOFF) ensured convergence to ∼0.1 meV per atom. Bulk hcp Ru was modeled using a 6 × 6 × 5 hexagonal supercell and an equivalent 6 × 3 × 5 orthohexagonal representation of the same optimized hcp lattice. The nine-layer Ru(0001) slabs used in the surface calculations were constructed from the optimized orthohexagonal representation and separated by a minimum 20 Å of vacuum. The PBE functional^41,42^ and D3(BJ) dispersion^43–45^ were used, with energy and force thresholds of 10^−7^ eV and 0.005 eV Å^−1^. Oxygen coverages *θ* were referenced to Ru(0001) hcp sites, where *θ* = 1 corresponds to a (1 × 1)O overlayer. Ordered phases were denoted using Wood's notation (*A* × *B*).^46^ A symmetry-adapted enumeration generated 174 unique O configurations from (6 × 6) (*θ* ≈ 0.03) to (1 × 1) (1.0 ML). Adsorption energetics and coverages were post-processed using ASE^47^ and NumPy,^48^ with automated Ru-layer detection and normalized O-counts following the methodology in ref. 8.1
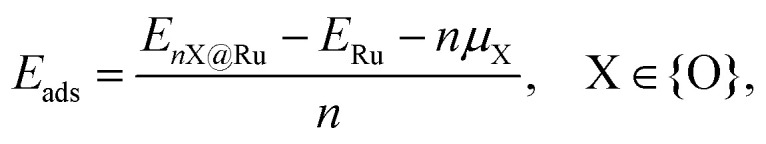
where *E*_ads_ is the mean adsorption energy per O atom, *E*_*n*X@Ru_ is the total energy of the Ru slab containing *n* adsorbed X atoms, *E*_Ru_ is the total energy of the corresponding clean Ru slab, *n* is the number of adsorbed atoms, and *μ*_X_ is the chemical potential of the adsorbed species. In this work, X = O and 
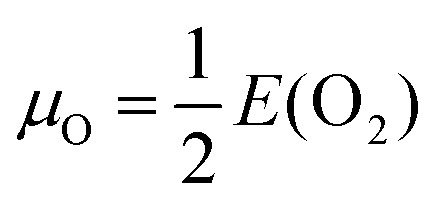
 from gas-phase calculations at the same level of theory.

For each O/Ru(0001) surface, SiH_4_ was positioned above candidate adsorption O-sites, Ru-sites, and mixed O–Ru—sites identified within 3.0 Å of the surface and filtered by local fingerprint uniqueness (radius 4.0 Å, *z*-window 3.0 Å). A minimum-image clustering scheme (link radius 3.0 Å) selected unique SiH_4_ configuration per connected region. The SiH_4_ molecules were placed 2.0 Å above the reference atom (H_base_/H_down_/Si) and shifted outward to maintain a 2.0 Å H–surface clearance.

SiH_4_ interaction energies were evaluated as

2 *E*_Interaction_(SiH_4_) = *E*_final_ − *E*_slab_ − *E*_SiH_4__,

where *E*_Interaction_(SiH_4_) is the interaction energy of SiH_4_ with the corresponding Ru or O/Ru slab, *E*_final_ is the total energy of the fully relaxed final structure after SiH_4_ placement and relaxation, *E*_slab_ is the total energy of the corresponding slab before SiH_4_ placement, and *E*_SiH_4__ is the total energy of isolated gas-phase SiH_4_ calculated at the same level of theory. For intact final-state SiH_4_, this quantity corresponds to a molecular adsorption energy. For final states in which one or more Si–H bonds are cleaved, the same expression describes the overall dissociative interaction energy after relaxation relative to gas-phase SiH_4_ and the corresponding slab. All H atoms originating from SiH_4_ are retained in the relaxed final-state structure; no separate gas-phase H_2_ or H_2_O products are included in this reference state. The resulting *E*_Interaction_ values were plotted as a function of *θ*_O_ and classified by final-state Si–H coordination (SiH_4_, SiH_3_, SiH_2_, SiH, Si). In this static 0 K PBE + D3(BJ) framework, dissociation denotes relaxation of an initially molecular SiH_4_ configuration to a dissociated local minimum on the potential-energy surface, without implying a calculated kinetic barrier or rate. Structure handling, database management, and plotting employed ASE, NumPy, and Matplotlib^49^; site sampling and fingerprinting followed the workflow described in our previous work.^8,50,51^

## 3 Results & discussion

### 3.1 Silicon deposition due to silane exposure

**Fig. 1:**
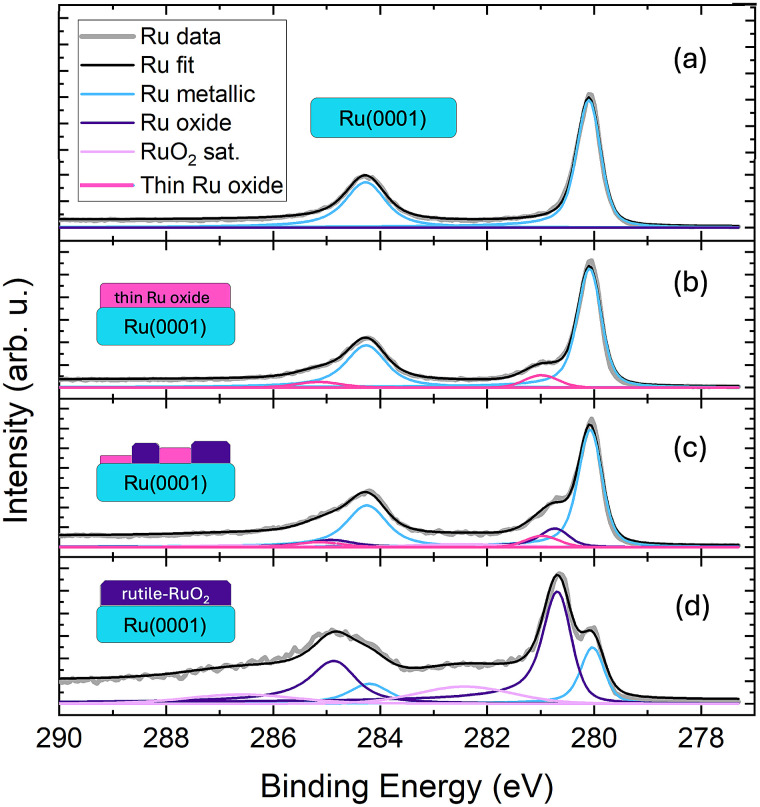
High-resolution XPS spectra of the Ru 3d region and illustrations of the phases in the surface region for pristine Ru(0001) (a), thin oxide (b), mixed oxide (c) and thick oxide (d). The individual components are drawn in blue for Ru metal, pink and deep purple for oxidized Ru^4+^ from thin and thick RuO_2_ layers, respectively and in lilac for the satellite of rutile RuO_2_.

As a starting point for the comparative study of silane adsorption, four different Ru-based surfaces were prepared: pristine Ru(0001) and Ru(0001) with the intermediate oxide (0.4 nm RuO_2_, referred to as “thin oxide”), with the bulk-like RuO_2_ in the rutile structure (4.2 nm, “thick oxide”), and with a mixture of the two oxide phases (equivalent thickness of 1.2 nm, “mixed oxide”). The thickness estimates are derived from the relative peak intensities of the Ru 3d XPS spectra shown in Fig. 1. The peak fits show the components corresponding to metallic Ru (280.06 eV, blue line), thin Ru oxide (280.97 eV, pink line), rutile RuO_2_ (280.69 eV, deep purple line), and the characteristic satellite peak for the rutile RuO_2_ phase (282.46 eV, purple line) peaks, with numbers provided at this precision to convey the fixed binding energy differences used in peak fitting. Literature values^52,53^ were used as starting points for the fits. The individual peak positions and shapes were adapted to match clear emerging features from the *in situ* spectra and were kept constant for all data sets. A complete overview of the fitting parameters, constraints, and component assignments used throughout the analysis is provided in the supplementary information. The different colors in the sketches next to the spectra illustrate the evolution of the oxide phase with thickness, with pink features representing the intermediate oxide and purple the rutile RuO_2_ phase. The coexistence of patches with both structures is inferred from the gradual increase in the relative peak area of the satellite features, which indicates an increasing contribution of the rutile RuO_2_ phase to the total oxide layer. This coexistence of two phases also means that the approximation of a homogeneous layer certainly does not hold for the mixed oxide, which is likely to consist of a surface of intermediate RuO_2_ with a small density of thicker patches of rutile RuO_2_. Thus, the thickness estimate of 1.2 nm should only be interpreted as an indication of an oxide containing both the thin and thick type of layer.

**Fig. 2:**
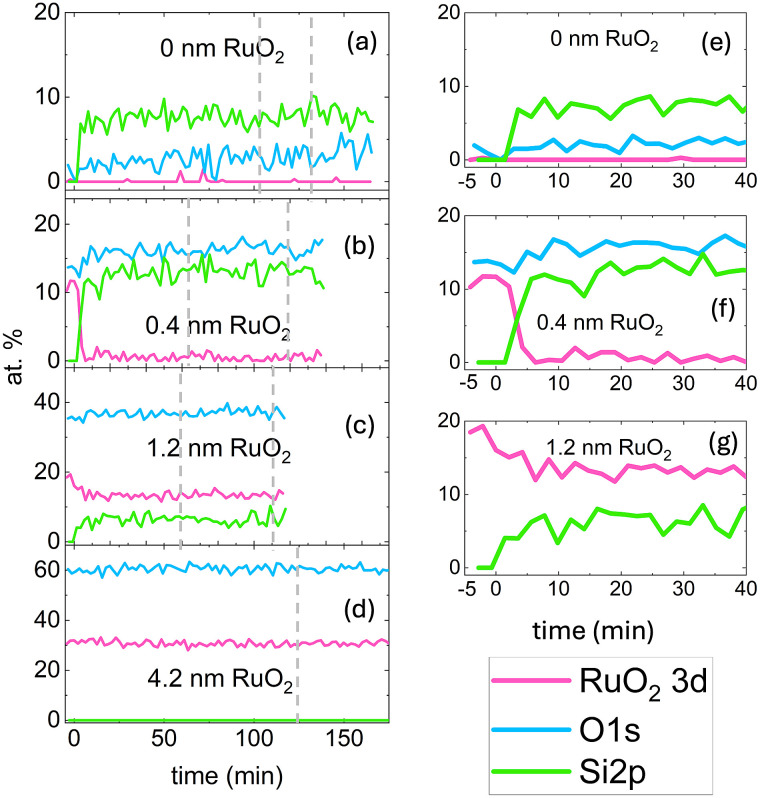
Evolution of the oxygen, silicon, and Ru^4+^ (RuO_2_) contents during exposure of (a) pristine Ru(0001) (0 nm RuO_2_), (b) Ru(0001) with thin Ru oxide (0.4 nm RuO_2_), (c) the mixed oxide (1.2 nm RuO_2_), and (d) the thick rutile ruthenium oxide, r-RuO_2_ (4.2 nm RuO_2_) to SiH_4_ pressures from 4 × 10^−8^ mbar to 4 × 10^−6^ mbar. The grey dashed lines indicate pressure increase steps by a factor of 10. The right column, (e), (f) and (g) panels, shows insets of the first minutes of exposure, illustrating the rapid growth of Si concomitant with a decrease of the Ru^4+^ content.

The evolution of the surface composition of the metallic and oxidized Ru(0001) was followed during exposure to SiH_4_, starting at equivalent pressures of approximately 4 × 10^−8^ mbar and increasing to 4 × 10^−6^ mbar after changes at low pressure had saturated. Fig. 2 shows the atomic percentage of Si 2p, O 1s and the Ru^4+^ content of the Ru 3d region as a function of exposure time to SiH_4_, with insets (e–g) in the right column zooming in on the changes in the initial phase of exposure. The times, at which the pressures were increased by a factor of 10, are indicated by grey lines. The respective initial and final compositions are summarized in Table 1. For pristine Ru (0 nm RuO_2_, Fig. 2(a) and (e)), the Si signal increases rapidly but the growth saturates quickly after less than ten minutes. The oxygen content increases gradually, but no signal of oxidized Ru or Si is observed, indicating the slow accumulation of adsorbed oxygen. When starting from the thin oxide (0.4 nm RuO_2_, Fig. 2(b) and (f)), Si deposition also occurs within the first ten minutes, similar to the metallic surface. At the same time, the Ru^4+^ content drops to zero, while the oxygen content is maintained and even slightly increases. The mixed oxide (1.2 nm RuO_2_, Fig. 2(c) and (g)) exhibits similar behavior, with fast growth of the Si signal but it saturates at a lower total Si coverage and exhibits a finite Ru^4+^ content after the deposition. For both oxides, the change in the Si content is more pronounced than the decrease of the Ru^4+^ content. In contrast to the metallic and mildly oxidized cases, the thick RuO_2_ layer (4.2 nm RuO_2_, Fig. 2(d)), does not present any discernible change upon SiH_4_ exposure in the *in situ* measurement despite a longer exposure than for the thin oxide. The second pressure increase performed after 190 minutes is not plotted but did not lead to any further change. Detailed Si 2p scans after the *in situ* experiment reveal a small fraction of Si (∼1% relative intensity), which we attribute to a small residual content of the intermediate oxide or extended defects such as step edges.

**Table 1:** Detailed composition of the different initial Ru surface preparations before and after silane exposure

	Initial	Final	Initial	Final	Initial	Final
	Ru^4+^ %	Ru^4+^ %	O %	O %	Si %	Si %
Pristine Ru	—	—	—	2	—	12
Thin oxide	10	—	11	16	—	13
Mixed oxide	17	13	35	36	—	8
Thick oxide	33	32	58	57	—	1

**Fig. 3:**
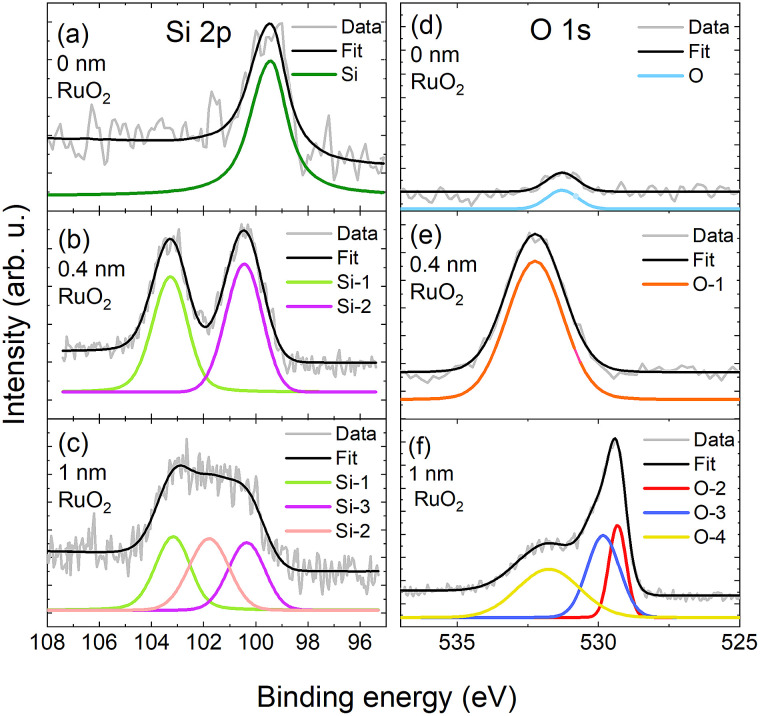
Si 2p and O 1s core level XPS spectra acquired after silane exposure for pristine Ru, thin oxide, and mixed oxide.

To gain insights into the underlying deposition process and the nature of the deposited species, the Si 2p and O 1s spectra taken after the silane exposure are examined in detail. Fig. 3(a) shows that on Ru metal, Si deposits as a single species, illustrated by a single Si 2p peak at 99.6 eV. The precise identification of the deposited Si species is not straightforward, as the characteristic XPS peaks, Ru 3d and Si 2p, show only subtle changes in peak position between the elemental materials and their compounds. In the XPS literature, the 2p binding energy of elemental Si^0^ ranges from 98.3 eV^54^ to a range of values above 99 eV,^55–58^ and even up to 100 eV for high doping levels.^59^ Next to elemental Si, the binding energies of Ru silicides have been reported to match the observed values, with Si 2p core levels ranging from 99.3 to 99.8 eV.^56,60,61^ A key limitation in comparing our system with previous studies is that they investigate Ru on Si, while we investigate Si on Ru, which presents different intermixing behaviors and possibly different Ru/Si ratios, which can be reflected in different surface chemistry. Work by van Vliet *et al.*^62^ showed shifts in the 2p binding energy with the Si content of thin layers, ranging from 99.4 eV for Si on Si to 99.7 eV for Ru_2_Si_3_ and 100.0 eV for RuSi films. These results were related to the plasmon peak of the silicides for their identification, but the weak signal and the overlap with stronger features from metallic Ru (3d and 4p), prevent us from using this method of assigning the Si species. Reports of intermixing upon deposition of Si on Ru at room temperature^63,64^ indicate that silicide formation is likely to occur also on the surfaces presented here.

The cases of the oxides are equally intriguing, but more complex. While there is clear contrast between the absence of Si deposition for the bulk-like rutile RuO_2_ and its rapid growth on the thin oxide, resolving the individual species is challenging. Two components are required to fit the Si 2p peak of the thin oxide in Fig. 3(b), which has distinct features at 100.4 eV and 103.2 eV. The respective O 1s peak after exposure in Fig. 3(e) is in agreement with a single species centered at 532.2 eV. The mixed oxide with elements of the thin oxide and the inert rutile RuO_2_ seems to behave like a superposition of the two in the time-dependent results shown previously, with deposition only occurring on the thin oxide. The detailed analysis of the spectrum in Fig. 3(c), however, presents three Si 2p peaks. In addition to the two peaks at 100.4 eV and 103.2 eV, an additional one is observed at 101.8 eV. Fitting the respective O 1s peak, shown in Fig. 3(f), also requires three components at 531.8 eV, 529.9 eV, and 529.3 eV.

**Fig. 4:**
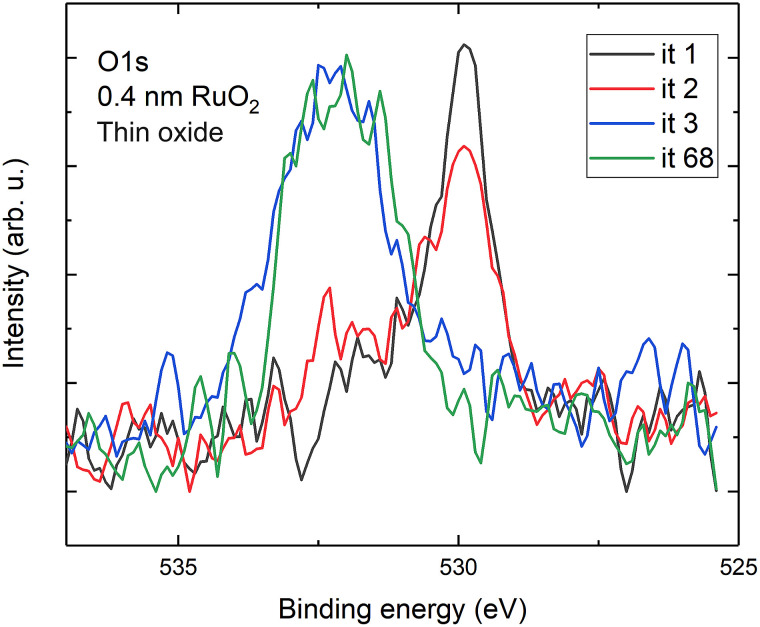
XPS spectra of the O 1s region of the thin oxide (0.4 nm) during iteration 1 (before SiH_4_), and 2, 3 and 68 of exposure to SiH_4_.

For the identification of the post-exposure species, it is essential to establish a link between the formation of the oxygen and silicon species and the modification of the Ru species at the surface, specifically the extinction of Ru^4+^ during the deposition of Si at the surface can clearly be resolved. To better understand the RuO_2_ compositional transformation upon Si deposition in the thin oxide, the *in situ* evolution of the oxygen species during silane exposure is analyzed in Fig. 4. Prior to silane exposure, iteration 1 finds the O 1s peak at 529.8 eV, as expected for Ru oxide.^52^ During iteration 2 silane gas is leaked in, which is reflected immediately in the O 1s signal by a lower intensity for the peak at 529.8 eV and the growth of a shoulder at 532.2 eV. Already in the next iteration approximately 2 minutes later, the feature at 532.2 eV becomes the dominant component of the O 1s peak. This broad peak remains unchanged within measurement error for the remaining 65 iterations. The difference in the O 1s peak binding energy showcases a complete change to a different surface species, while the time resolution establishes a causal link to the interaction with silane. The binding energy position after silane exposure is characteristic for Si oxides rather than Ru oxides, indicating that the main bonding partner of the oxygen changed to become Si. Exposure of the thin Ru oxide to silane thus not only leads to Si deposition, but triggers a full transformation of the thin oxide layer into an oxidized Si compound.

From the oxygen spectra and the high-binding energy component of Si, it seems straightforward to assign the new surface species as SiO_2_. Typical Si 2p binding energies for SiO_2_ ranging from 103.2 eV^54^ and 103.3 eV^65,66^ to 103.9 eV^57^ allow the conclusion that the nominal oxidation state of the high-binding energy species is closest to Si^4+^. The Si peaks at lower binding energies, however, correspond neither to Si^4+^ nor to silicides. They are found in the range of silicon's sub-oxides, reported at binding energies of 101 eV^54^ or 101.3 eV^56^ for Si^1+^ and 101.8 eV for Si^2+^.^67^ The coexistence of multiple Si 2p components may reflect different Si–O–Ru bonding environments, similar to the configurations suggested for ultrathin Si oxide on Ru(0001) by Kremer *et al.*^68^ The characteristic features of the ultrathin oxide layer, SiO_4_ tetrahedra, are also a common motif in silicates. While no XPS studies of Ru silicates were found, Si binding energies in other silicates have been reported in a range of 101.3–102.7 eV,^55,69–71^ which is consistent with the intermediate binding energies observed upon silane exposure. This binding energy range agrees with the additional peak observed for the mixed oxide, possibly indicating the coexistence of silicates with different Ru contents. This is plausible since we expect the mixed oxide to consist of patches of intermediate oxide and RuO_2_ in rutile structure, providing reactive boundary sites of the two different oxides, in addition to the active sites on the pure thin oxide. In the absence of spectroscopic literature on ruthenium silicate, assigning the sub-oxides of Si to silicate-like structures, however, remains a speculation.

### 3.2 Origin of the different activity upon surface oxidation

**Fig. 5:**
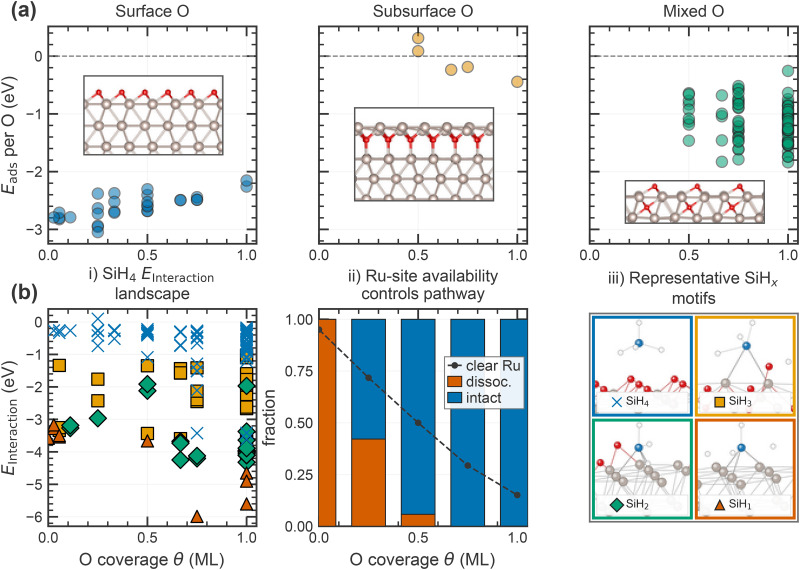
Panel (a) shows the adsorption energy per O atom as a function of O coverage up to one monolayer, resolved into surface, subsurface and mixed O configurations. The atomistic insets illustrate representative local O motifs, and these O/Ru surfaces form the starting configurations for the subsequent silane adsorption shown in panel (b). Panel (b)(i) shows the full SiH_4_ interaction-energy landscape without filtering by the energy of the parent O/Ru surface. The points are resolved by the final-state Si–H coordination after structural relaxation, with the corresponding SiH_*x*_ motifs shown in panel (b)(iii). Panel (b)(ii) summarizes the final-state pathway in 0.25 ML coverage bins after correcting for both the thermodynamic weight of the parent O/Ru surface and the local site degeneracy. For each coverage bin and parent surface, the lowest-energy relaxed final state in each channel is used: SiH_*x*_ final states with *x* < 4 and no Si–O bond are assigned to Ru-assisted dissociation, while intact SiH_4_ final states are assigned to molecular adsorption. The dissociation channel is weighted by the number of clear Ru sites, while the intact channel is weighted by the number of O-blocked sites; the dashed line indicates the corresponding clear-Ru-site fraction. In all panels with atomic configurations Ru are represented by silver, O by red, Si by blue and H by white spheres.

The different nature of the Si species formed on each surface termination, together with the transformation of the Ru oxide layer upon silicon deposition, raises the question whether the interaction with silane is comparable for the different surface configurations. To determine the effect of surface oxygen on the deposition mechanism, the adsorption of SiH_4_ on Ru metal surfaces with different coverages (*θ* ∈[0,1] ML) and configurations of oxygen was modeled using DFT calculations. Fig. 5 provides an overview of the configurations considered, illustrating that the interaction of SiH_4_ on Ru(0001) spans a continuum from weak physisorption to dissociative chemisorption, governed by the local availability of Ru surface sites. On the clean surface, SiH_4_ dissociates spontaneously to Ru–SiH + 3 × Ru–H; the Si fragment occupies an hcp hollow, and the H atoms relax to adjacent top, bridge, or hollow sites. Increasing O coverage progressively passivates Ru and suppresses dissociation. As shown in Fig. 5a, O adsorption stabilizes ordered hcp overlayers, while mixed or sub-surface configurations are disfavoured at low *θ* but become increasingly favoured beyond ∼0.5 ML, producing the broad band near −1.5 eV.

For SiH_4_ adsorption (Fig. 5b), close-packed O overlayers—locally nearest-neighbour (NN) complete structures such as the (1 × 1) phase—block Ru sites and enforce physisorption. When the O layer is more open or irregular (striped or patch-like domains), SiH_4_ dehydrogenates to SiH_*x*_ fragments (*x* = 1–3). Intermediate states (SiH_4_/SiH_3_/SiH_2_) appear where O crowding or surface rumpling limits access to multiple Ru neighbours—more strongly bound than physisorbed molecules but short of full Si–H scission. The energetic ordering is dictated by H termination: at low *θ*, H remains bound as Ru–H on top/bridge sites; at higher *θ*, nearby O accepts H to form surface OH, which markedly deepens the adsorption well. Because *θ* includes both surface and sub-surface O, dissociation can persist near *θ* ≈ 1 ML whenever a fraction of O resides below the surface, leaving Ru surface sites.

Dissociative adsorption progressively consumes the remaining reactive Ru sites. Initial SiH_4_ molecules dissociate at exposed Ru hollows, depositing Si at hcp positions and passivating adjacent top or bridge sites through Ru–H or, where O is present, OH formation. Each dissociation therefore reduces the local density of available Ru sites, and once the nearest-neighbour ring around an adsorption site becomes filled (NN-complete), subsequent SiH_4_ adsorption is frustrated. Sub-surface O remains energetically unfavourable and does not influence this process. The reaction thus becomes self-limiting: SiH_4_ dissociation ceases as the surface approaches O-, Si- and H-induced site saturation, providing the atomistic origin of the rapid saturation of Si observed in the experiment.

**Fig. 6:**
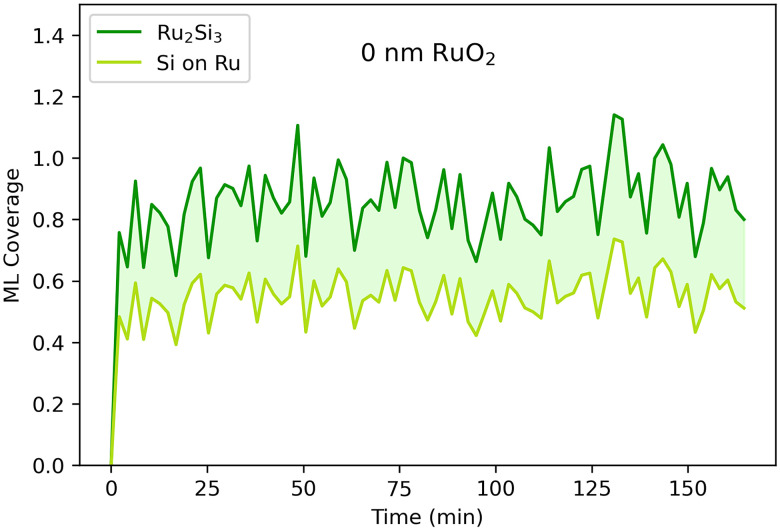
Comparison of the time-dependent coverage of Si-containing layers calculated from the surface Si content for two different models of the deposited compound. In the thinnest case, a monolayer of Si atoms on Ru (in light green), growth saturates at 0.55 ML, whereas Ru_2_*Si*_3_ silicide grows to about 0.86 ML (in dark green). The shaded area corresponds to coverages achieved for hypothetical layers with intermediate Si contents and electron attenuation.

The highly efficient dissociation predicted by DFT together with the quick saturation observed in experiment call for a quantification of the deposited amount and the experimental sticking coefficient. To relate the saturation density to a surface coverage, we approximate the coverage of the Si-containing layer by monolayers (ML) of constant thickness, calculated from the relative XPS intensities of the Si 2p and Ru 3d. Since the estimated coverage will vary with the deposited compound, we compare two different configurations of Si-containing layers at the surface as thin and thick approximation of the grown layer. In the thin limit, all Si is concentrated in a monolayer of Si atoms adsorbed at the surface with an estimated thickness of 0.17 nm, derived from the layer spacing reported from DFT.^8^ In the thicker approximation, the Si forms a compound with Ru and spreads over a larger thickness, selected to be a layer of the stable silicide Ru_2_Si_3_. For this case the thickness of 1 ML is taken as 0.28 nm, corresponding to one fourth of the unit cell's *c*-axis. This value reflects the thinnest layer that preserves the stoichiometry of the silicide.^72^ The comparison of the two cases is displayed in Fig. 6 and shows that the saturation coverage of Si on pristine Ru is between 0.55 ML (pure Si) and 0.86 ML of Ru_2_Si_3_. In the context of the DFT results, this coverage is high, demonstrating that hydrogen does not block sites adjacent to SiH_4_ dissociation permanently, but is mobile at the conditions of the experiment. This mobility keeps sites for dissociation available until a saturation coverage of Si is reached. The blocking of Ru sites by oxygen predicted by DFT suggests a different possible pathway, in which oxygen also competes for adsorption sites with silane to lower the total Si coverage.

Since SiH_4_ decomposes readily in the presence of Ru sites, the initial sticking coefficient of the molecule is expected to be reflected in the rate of Si deposition. The sticking coefficient can thus be directly approximated from the time-dependent growth curves of the Si-containing layer. For an impingement rate of silane of Γ(SiH_4_) = 1.1 × 10^13^ molecules cm^−2^ s^−1^ and a site density of 1.75 × 10^15^atoms per cm^2^, we expect the molecules to have interacted with each surface atom after approximately 159 seconds. Only if all these interactions lead to the dissociation of silane and deposition of silicon, a full monolayer will grow within this short time. For a layer of Ru_2_Si_3_, the number of Si atoms per surface area is 1.2 × 10^15^atoms per cm^2^ (in the coarse approximation of a 2.8 Å thick slice of an orthorhombic Ru_2_Si_3_ unit cell cut perpendicular to the *c*-axis). The experimental observation of a saturation coverage close to a full layer after less than five minutes of exposure thus demonstrates that the probability of dissociative sticking of silane is higher than 36%. Within the experimental time resolution, it can not be excluded that the coefficient is even closer to unity. After this initial phase of fast deposition, no further growth is observed within experimental accuracy, reflecting the self-limiting character of the deposition and the consumption of the active Ru sites in the process. This saturation behavior suggests the growth of a complete layer of electronically different material, which would be consistent with the growth of a silicide approaching a coverage close to a monolayer. However, the observations could also be explained by other models of growth and site blocking, for example the case of Si atoms covering Ru, which is shown to inhibit further reactivity towards Si deposition from silane by our calculations. The presence of adsorbed oxygen or hydrogen could present an explanation why the growth terminates at sub-monolayer coverages.

On the surface covered with a thin or mixed oxide, the deposition kinetics is similar, whereas no deposition occurs on the rutile RuO_2_ layer beyond a very small content that is likely caused by reactive surface defects or gas-phase activation by electrons. The fast initial growth on the thin oxide is followed by saturation at comparable coverages and time scales to the metal surface. The average sticking coefficient on the thin oxide is thus similar to that of the metal surface, indicating a comparable density of active sites. According to the DFT calculations, however, dissociative adsorption is not expected to occur on Ru sites with oxygen adsorbates. This would only leave a small number of potential active sites on an ordered oxidized surface and would significantly alter the Si growth kinetics. Moreover, the total amount of deposited Si would decrease drastically, which is in contrast to the experimental results. Alternative explanations, such as the coexistence of oxide patches with large areas of Ru metal are not consistent with the different Si 2p binding energies for silane decomposition over Ru metal and thin Ru oxide. The peak positions of Si at the oxidized Ru surfaces demonstrate that all the observable Si interacts with oxygen, likely forming Si–O–Ru bonds and potentially also binary silicon oxides. The strong interaction of the deposited Si across the entire oxidized surface is further highlighted by the observation that all Ru in the thin oxide layer is reduced after silane exposure. However, according to the DFT calculations, oxygen adsorption on an ordered Ru surface deactivates the metal sites for silane decomposition, leading to the conclusion that the thin oxide layer deviates from a Ru surface with adsorbed and incorporated oxygen. While the simulation of layers with entirely different structural and electronic properties is beyond the scope of this study, the observed behavior could also be explained by a high level of disorder, in agreement with literature reports of the intermediate oxide.^73^ The high density of defect sites on a strongly disordered surface could provide ample opportunity for the adsorption and decomposition of silane, leaving behind Si and hydrogen, which readily bond to surface oxygen.

The proposed role of disorder and defects also prompts the question whether the sample preparation, in particular roughening by sputtering, influences the observed result. While the observation of sharp diffraction spots in LEED patterns after preparation of the pristine metal surface (see Supporting Information) demonstrates that large patches of the surface are ordered, a remaining increase in the density of defects and steps cannot be excluded. A more defective metal surface would likely also translate to more defects in the thin oxide layer prepared by annealing in oxygen. The observation of rapid deposition of Si up to a saturation coverage close to a full layer, however, highlights that also the defect-free Ru(0001) surface efficiently splits SiH_4_, in agreement with the DFT results. Similarly, the deposition on the thin oxide layer is rapid with a high sticking coefficient, indicating a very high defect density that is more likely intrinsic to the initial oxide formed before converting to the stable rutile structure. This interpretation agrees well with the absence of Si deposition on the thick oxide layer, suggesting that defects do not play a role in silane decomposition after this structural change.

In addition to the deposition of Si described above, the decomposition of SiH_4_ also liberates four hydrogen atoms per molecule. At the pristine Ru(0001) surface, the Ru metal lattice provides adsorption sites for hydrogen atoms, yielding Ru–SiH_(4−*n*)_ + *n*Ru–H. While hydrogen atoms are predicted to block sites for further silane adsorption according to the DFT calculation, reflecting the energy at 0 K, this effect is likely negligible thanks to the high mobility of adsorbed hydrogen at room temperature. Moreover, the rate of recombination and desorption of hydrogen is expected to be significant at the time scale and temperature of the experiment, based on temperature programmed desorption studies^74^ whereas solubility in bulk Ru has been reported to be low.^75^ Second order desorption peaks reaching close to 300 K for high coverages indicate a finite desorption rate should be expected at room temperature. High-coverage regions of hydrogen would thus result in efficient recombinative desorption from Ru metal, leaving only a low density of hydrogen atoms with long residence time. Upon dissociation of silane over thin Ru oxide, on the other hand, the released hydrogen likely forms hydroxyl groups, which are stable at room temperature. Accordingly, we considered the presence of an O 1s component at binding energies previously attributed to OH species on Ru surfaces.^76^ Indeed, the two spectra acquired during the main increase of Si intensity (iterations 2 and 3 in Fig. 4) exhibit additional intensity within this energy range. However, the transient nature of this feature, combined with the ambiguous composition and structure of the Ru–Si–O phase, precludes assigning definitive significance to its observation. Our results do not allow for assigning the detailed reaction steps with certainty, and pathways beyond adsorption and dissociation remain speculative. Adsorption on undercoordinated Ru sites, yielding Ru–SiH_4−*n*_ + *n*Ru–O–OH is considered a likely first step. Among multiple potential options with comparable experimental signature, the formation of SiRu_2_O_4_ + 2Ru(OH)_2_ would for example fulfil mass and charge balance of the entire layer and result in reduced Ru. Nevertheless, important uncertainties remain, for example whether the layer would remain intact at this high hydrogen concentration and whether hydroxyl groups would be stable at room temperature in the Si–Ru–O compound.

**Fig. 7:**
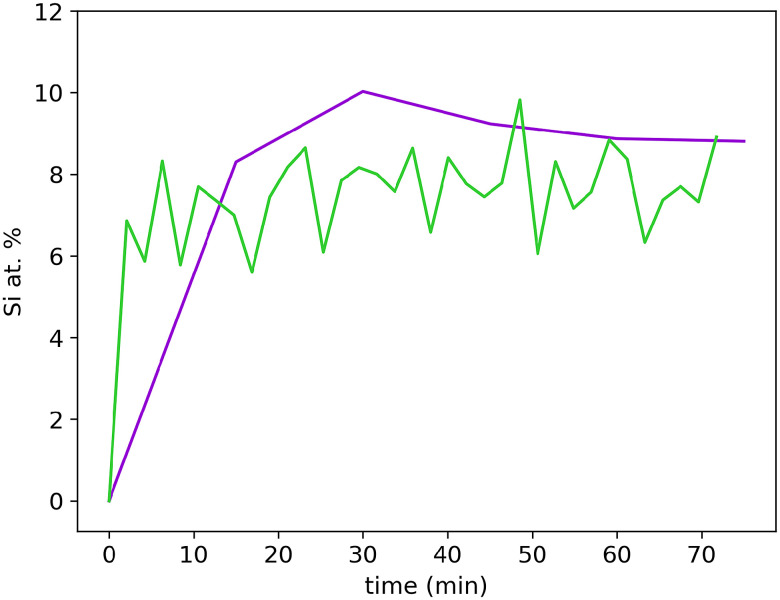
Evolution of silicon atomic percentage throughout the exposure of Ru (0 nm RuO_2_) to SiH_4_ in Ar with (green) and without (lilac) simultaneous irradiation by X-rays at the same silane pressure.

The observation of the high sticking coefficient of silane prompted an experimental test of the interference of the *in situ* measurement with its outcome by activating the silane molecules. Fig. 7 shows a comparison between the silane exposure to pristine Ru with (green) and without (lilac) simultaneous exposure to the Al Kα X-rays used by the spectroscopy tool. For the two data sets a comparable growth trend and the same final Si atomic percentage were observed, regardless of X-ray exposure during the experiment. Most importantly, the fast deposition within the first minutes of exposure was also observed without simultaneous exposure to X-rays. These results demonstrate that additional activation by the X-rays does not contribute decisively to the high sticking coefficient or the resulting Si deposition.

## 4 Conclusions

In summary, the interaction of Ru surfaces with silane changes drastically between surfaces terminated with Ru metal, the thin intermediate Ru oxide, and the bulk-like rutile RuO_2_. Silane readily decomposes on Ru metal and the intermediate oxide with a high sticking coefficient. In both cases, the growth of Si is self-limiting and terminates at coverages close to a full layer of Ru–Si compounds. The surface species formed upon deposition, however, is different. On Ru metal, elemental Si or Ru silicide is observed as a direct result of the spontaneous dissociation of silane. Upon silane exposure of the thin Ru oxide layer, oxidized Si species form, which are reminiscent of silicates and SiO_2_, whereas Ru changes to a fully reduced state. The deposition is attributed to splitting of silane at active sites arising from disorder in the oxide layer. In contrast to the thin oxide, a 4 nm thick layer of rutile RuO_2_ proves to be fully inert towards silane decomposition and preserves its original surface composition throughout several hours of experiments. These results demonstrate that the oxidation state and structure of the Ru-containing surface layer is decisive for the reactive decomposition of growth precursor molecules using the example of silane.

## Conflicts of interest

There are no conflicts of interest to declare.

## Supplementary Material

CP-028-D6CP01417H-s001

## Data Availability

The raw/processed data required to reproduce these findings are publicly available *via* a Zenodo repository at the DOI: https://doi.org/10.5281/zenodo.19598722. Supplementary information (SI) is available. See DOI: https://doi.org/10.1039/d6cp01417h.
