# Supplementary material for: The effect of ruthenium oxidation on the decomposition of SiH4
Source: Phys Chem Chem Phys. 2026 Jun 16;28(27):16709–20. doi: 10.1039/d6cp01417h (PMC13288707; doi:10.1039/d6cp01417h)
Supplement: CP-028-D6CP01417H-s001 [file CP-028-D6CP01417H-s001.pdf]

Supplementary Information (SI) for Physical Chemistry Chemical Physics.
This journal is © the Owner Societies 2026

# Supporting information

## The effect of ruthenium oxidation on the decomposition of $SiH_4$

Ester Pérez Penco[1], Jonathon Cottom[1,2], Emilia Olsson[1,2], Roland Bliem[1,3]*

[1] *Advanced Research Center for Nanolithography (ARCNL), Science Park 106, 1098 XG Amsterdam, The Netherlands*

[2] *Institute of Theoretical Physics, Institute of Physics, University of Amsterdam, Science Park 904, 1098 XH Amsterdam, The Netherlands*

[3] *Van der Waals–Zeeman Institute, Institute of Physics, University of Amsterdam, Science Park 904, 1098 XH Amsterdam, The Netherlands*

**Corresponding author:* r.bliem@arcnl.nl

**1. XPS peak fitting procedure** – this section provides the details of the fitting procedure employed to obtain the results presented in the manuscript.

For the Ru 3d region, a Shirley background was used. The energy difference and area ratio (Ru $3d_{5/2}$:Ru $3d_{3/2}$=1.5) of the spin-orbit-split components of Ru 3d was constrained for all fits. The fitting parameters were optimized using a reference spectrum acquired from clean Ru(0001). Upon partial and near-complete oxidation of the surface, additional Ru oxide components were introduced while maintaining the same relative constraints between the 5/2 and 3/2 components. For the metallic and oxide Ru 3d components, Doniach–Šunjić–Gaussian (DS-G) line shapes were used to account for the metallic character of Ru and $RuO_2$. An increased Lorentzian width was allowed for the Ru $3d_{3/2}$ component to account for Coster–Kronig broadening. The thin oxide components and satellite features in the Ru 3d region were fitted using Voigt line shapes. The corresponding satellite peak areas were constrained to also maintain a 1.5 area ratio between the Ru $3d_{5/2}$ and Ru $3d_{3/2}$ components. The area ratio of the satellite peak and the main core level peak was determined from calibration measurements on reference oxide spectra and subsequently kept constant for all oxidized Ru components throughout the fitting procedure. For the pristine Ru and the thick oxide, the fit in Table I was used. For pristine Ru, the area of the oxide-related components decreased to zero, reflecting the absence of oxide species. The electronically different thin oxide required different fit parameters, characterized by a small shift of the oxidized Ru core level and the absence of the characteristic satellite of rutile $RuO_2$. The fit parameters are provided in Table II. The mixed oxide was fitted accounting for the coexistence of thin oxide and thicker ($RuO_2$) oxide components.

For both, O 1s and Si 2p spectra, the focus was the determination of the total area of peaks emerging at a priori unknown binding energies and peak shapes. No constraints were applied to those peaks other than the energy difference and peak area ratio of the spin-orbit split for the Si 2p components. For all Si 2p spectra, a Shirley background was used. The individual Si components were fitted using Voigt doublets, reflecting the spin-orbit split components of Si $2p_{3/2}$ and Si $2p_{1/2}$ with a fixed area ratio of 2:1 and a binding energy difference of 0.6 eV between the two components. The reported binding energies (BEs) are given by the peak position resulting from summing up the components of the fitted double Voigt. The O1s spectra were fitted using Voigt peak shapes.

| Pristine Ru & Thick $RuO_2$ | Ru $3d_{5/2}$ | Ru $3d_{3/2}$ | $RuO_2$ $3d_{5/2}$ | $RuO_2$ $3d_{3/2}$ | $RuO_2$ $3d_{5/2}$ satellite | $RuO_2$ $3d_{3/2}$ satellite |
|---|---|---|---|---|---|---|
| Peak shape | DS-G | DS-G | DS-G | DS-G | Voigt | Voigt |
| BE (eV) | 280.1 | 284.3 (BE Ru $3d_{5/2}$+4.2) | 280.7 (BE Ru $3d_{5/2}$+0.6) | 284.9 (BE $RuO_2$ $3d_{5/2}$+4.2) | 282.5 (BE Ru $3d_{5/2}$+2.4) | 286.7 (BE $RuO_2$ $3d_{5/2}$ satellite + 4.2) |
| Lorentzian width (eV) | 0.190 | 0.600 | 0.200 | 0.700 | 0.200 | 0.700 |
| Lorentzian asymmetry (eV) | 0.045 | 0.035 | 0.130 | 0.114 | - | - |
| Gaussian width (eV) | 0.400 | 0.478 | 0.467 | 0.467 | 2.000 | 2.000 |

Table SI: XPS fitting parameters for pristine Ru and $RuO_2$. BE refers to the fitted binding energy of the peak component.

| Thin oxide | Ru $3d_{5/2}$ | Ru $3d_{3/2}$ | $RuO_2$ $3d_{5/2}$ | $RuO_2$ $3d_{3/2}$ | $RuO_2$ $3d_{5/2}$ satellite | $RuO_2$ $3d_{3/2}$ satellite |
|---|---|---|---|---|---|---|
| Peak shape | DS-G | DS-G | Voigt | Voigt | Not present | Not present |
| BE (eV) | 280.1 | 284.3 (BE Ru $3d_{5/2}$+4.2) | 281.0 (BE Ru $3d_{5/2}$+0.9) | 285.2 (BE $RuO_2$ $3d_{5/2}$+4.2) | - | - |
| Lorentzian width (eV) | 0.190 | 0.600 | 0.001 | 0.238 | - | - |
| Lorentzian asymmetry (eV) | 0.045 | 0.035 | - | - | - | - |
| Gaussian width (eV) | 0.400 | 0.478 | 0.740 | 0.848 | - | - |

Table SII: XPS fitting parameters for thin $RuO_2$. BE refers to the fitted binding energy of the peak.

| Panel Fig. 3 | Si Peak | BE apparent max. (eV) | BE Si $2p_{3/2}$ (eV) | Lorentzian width (eV) | Gaussian width (eV) | Type |
|---|---|---|---|---|---|---|
| (a) - Pristine | Si | 99.6 | 99.6 | 1.51 | 0 | 2×Voigt |
| (b) - Thin oxide | Si-1 | 103.2 | 103 | 0.32 | 1.20 | 2×Voigt |
| (b) - Thin oxide | Si-2 | 100.4 | 100.2 | 0 | 1.49 | 2×Voigt |
| (c) - Mixed oxide | Si-1 | 103.2 | 103 | 0.32 | 1.20 | 2×Voigt |
| (c) - Mixed oxide | Si-2 | 101.8 | 101.7 | 0 | 1.49 | 2×Voigt |
| (c) - Mixed oxide | Si-3 | 100.4 | 100.2 | 0 | 1.49 | 2×Voigt |

Table SIII: XPS fitting parameters for Si used in Figure 3 of the manuscript. "BE apparent max." refers to the binding energy of maximum intensity for the sum of the Si $2p_{3/2}$ and Si $2p_{1/2}$ peaks spaced 0.6 eV, while BE Si $2p_{3/2}$ is the binding energy of the first component only.

| Panel Fig. 3 | O Peak | BE (eV) | Lorentzian width (eV) | Gaussian width (eV) | Type |
|---|---|---|---|---|---|
| (a) - Pristine | O | 531.3 | 0 | 1.38 | Voigt |
| (b) - Thin oxide | O-1 | 532.2 | 0 | 2.36 | Voigt |
| (c) - Mixed oxide | O-1 | 529.3 | 0 | 0.72 | Voigt |
| (c) - Mixed oxide | O-2 | 529.9 | 0 | 1.34 | Voigt |
| (c) - Mixed oxide | O-3 | 531.8 | 0 | 2.59 | Voigt |

Table SIV: XPS fitting parameters for O used in Figure 3 of the manuscript.

**2. LEED patterns** - To verify the crystallographic orientation and surface order of the sample, low-energy electron diffraction (LEED) measurements were performed following the standard cleaning procedure described in the manuscript. The resulting diffraction pattern exhibits the expected hexagonal symmetry of a well-ordered Ru(0001) surface, confirming the surface orientation and long-range order.

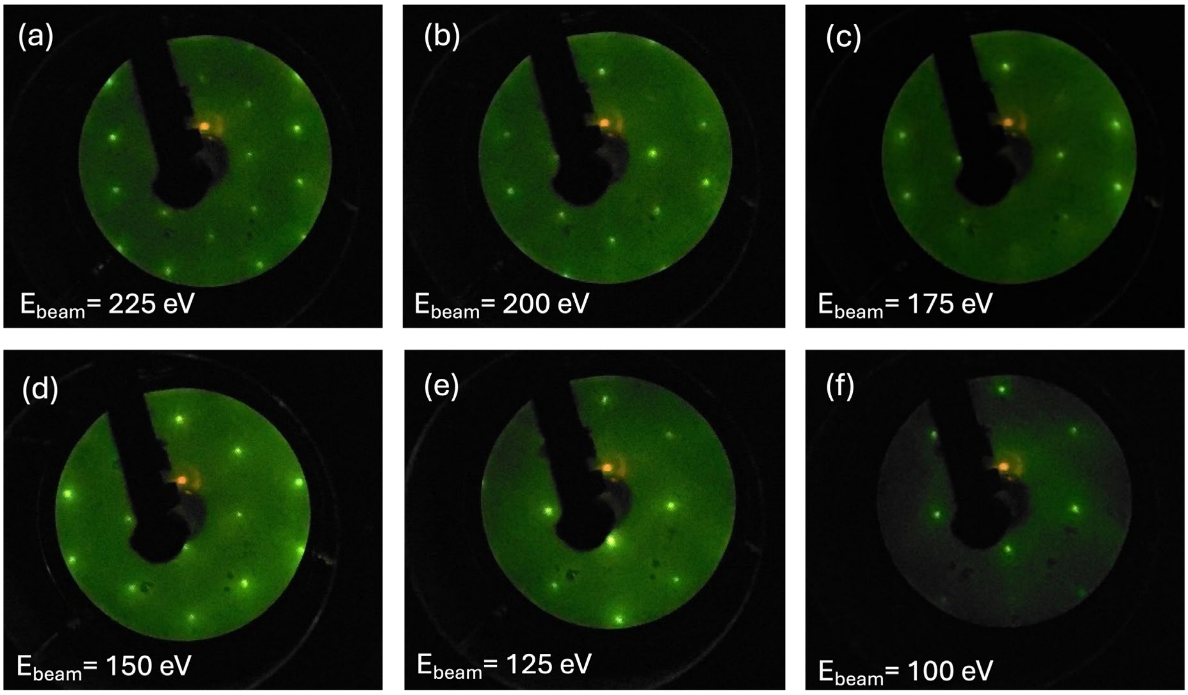


Figure S1: LEED patterns of Ru(0001) for varying beam energy.
